# Micro-expression recognition model based on TV-L1 optical flow method and improved ShuffleNet

**DOI:** 10.1038/s41598-022-21738-8

**Published:** 2022-10-20

**Authors:** Yanju Liu, Yange Li, Xinhan Yi, Zuojin Hu, Huiyu Zhang, Yanzhong Liu

**Affiliations:** 1 School of Mathematics and Information Science, Nanjing Normal University of Special Education, Nanjing, 210038 China; 2 School of Computer and Control Engineering, Qiqihar University, Qiqihar, 161000 China

**Keywords:** Computer science, Information technology

## Abstract

Micro-expression is a kind of facial action that reflects the real emotional state of a person, and has high objectivity in emotion detection. Therefore, micro-expression recognition has become one of the research hotspots in the field of computer vision in recent years. Research with neural networks with convolutional structure is still one of the main methods of recognition. This method has the advantage of high operational efficiency and low computational complexity, but the disadvantage is its localization of feature extraction. In recent years, there are more and more plug-and-play self-attentive modules being used in convolutional neural networks to improve the ability of the model to extract global features of the samples. In this paper, we propose the ShuffleNet model combined with a miniature self-attentive module, which has only 1.53 million training parameters. First, the start frame and vertex frame of each sample will be taken out, and its TV-L1 optical flow features will be extracted. After that, the optical flow features are fed into the model for pre-training. Finally, the weights obtained from the pre-training are used as initialization weights for the model to train the complete micro-expression samples and classify them by the SVM classifier. To evaluate the effectiveness of the method, it was trained and tested on a composite dataset consisting of CASMEII, SMIC, and SAMM, and the model achieved competitive results compared to state-of-the-art methods through cross-validation of leave-one-out subjects.

## Introduction

Whether consciously or unconsciously, facial micro-expressions are the result of hiding one's true emotions. A micro-expression is a facial expression that is rapid and unconscious. Micro-expression recognition has many potential applications in a variety of fields^1^, including the police services and lies detection^2^, teaching assistance^3^ and clinical diagnosis^4^. Even after training with micro-expression training tools^5^, humans find it difficult to accurately recognize micro-expressions due to the subtlety and transient nature of facial movements. As a result, using computers to recognize micro-expressions is a more effective solution. During the past several years, micro-expression recognition tasks have developed tremendously as benchmark datasets (e.g., CASME II^6^, SMIC^7^, SAMM^8^, MMEW^9^) and computer vision techniques have been developed.

By using deep learning for micro-expression recognition, the recognition accuracy has also improved significantly compared to the initial traditional approach of manually extracting facial features. Generally, manual feature extraction techniques can be divided into two broad categories: feature extraction based on variations in facial texture and feature extraction based on variations in facial light. Several researchers have used local binary patterns on three orthogonal planes (LBP-TOP)^10,11^ descriptors to describe changes in facial dynamic texture. In order to optimize or improve the basic LBP-TOP for better extraction of facial texture variations, the second-order Gaussian jets have been employed on LBP-TOP^12^, LBP six intersection points (LBP-SIP)^13^, space–time LBP (STLBP)^14^ and space–time completed local quantized patterns (STCLQP)^15^. For facial light variation analysis, directional mean optical flow (MDMO)^16^, bi-weighted directional optical flow (Bi-WOOF)^17^ and fuzzy optical flow directional histograms (FHOFO)^18^ are all capable of obtaining sufficient light variation information. Li et al.^19^ proposed a deep multi-task learning method to segment facial signs and then combine them with HOOF features to evaluate expression classes by determining the direction of facial muscle movements. A classifier is constructed after the features have been extracted, and the most commonly used classifiers are support vector machines and random forests. Traditional manual extraction methods have improved the capability of the human mind to recognize micro-expressions, but they are still unable to handle the diversity of facial variations. By contrast, deep learning methods are capable of obtaining high quality features automatically, even if their characteristics are not fully understood. In order to recognize micro-expressions, some deep learning models have been used, such as pre-trained CNNs like OFFApexNet^20^, TSCNN^21^, and CNNs combined with long and short-term memory^22^. As a result of this end-to-end approach, these deep methods have produced state-of-the-art results on several datasets by modeling the spatio-temporal variation of faces, training the extracted features, and building classifiers autonomously. In contrast, deep network models based on convolutional structures suffer from local feature extraction, which makes it difficult to extract global features from samples. In recent image classification research, vision transformer (ViT)^23^ based on self-attentive mechanism has become increasingly popular, which has the advantage of extracting global features and has now become another major solution to the image classification problem. The original ViT divides the image into consecutive non-overlapping blocks, and then uses multi-headed self-attention in the transformer to learn features between the blocks. Its disadvantage is that it increases the complexity of operations and reduces the efficiency of operations. However, conventional ViT models have a large number of parameters and are difficult to run efficiently, so some researchers have proposed plug-and-play ViT modules that can be easily integrated into convolutional neural networks to provide local and global feature extraction capabilities to the models.

A composite micro-expression recognition model is proposed in this paper based on the CBAM module, the improved ViT module, and ShuffleNetV2. The first step in preprocessing is to extract the TV-L1 optical flow^24^ features of the samples for pretraining, followed by face alignment and masking. By preprocessing the samples, redundant features in the composite ShuffleNetV2 model can be reduced during training, thereby reducing memory and computing requirements, and improving the accuracy of recognition. As part of the training process, optical flow features are fed into the model, and the weights of the down sampled partial layers are retained when the model is most accurate, training it into an excellent feature extractor. In the final training, the weights of the layers are loaded and trained. Our main contributions are as follows:

- We pre-train the model with optical flow features to make it easier to capture the micro-movements of facial micro-expressions.
- We integrate the optimized plug-and-play ViT module and the CBAM module into the efficient ShuffleNetV2 model separately, so that global features can be extracted without any loss of efficiency.
- We demonstrate with extensive experiments that the proposed method is still competitive with state-of-the-art methods.

The structure of this paper is as follows. "Related works" section analyzes the relevant research on micro-expression recognition in recent years. "Micro-expression recognition model design" section describes the model proposed in this paper in detail, including data preprocessing, TV-L1 optical flow features, CBAM module, improved ViT module and ShuffleNetV2 model. Experimental results and performance evaluation are given in "Experiments" section. Finally, "Conclusion and future work" section summarizes the whole paper and proposes future research work.

## Related works

Although the methods based on manual feature extraction in the introduction take full advantage of the spatiotemporal variation of facial skin texture, they are unable to describe the sample structure, cannot distinguish the relationship between high-dimensional features, and do not have the computational power to meet the demands of real-time analysis. The accuracy of the above microexpression recognition methods is not significantly improved as far as accuracy is concerned. As a result of recent research, deep learning methods have been considered one of the most effective methods of learning visual features. Neural networks have been widely used to process images and classify them. With their end-to-end models, researchers are not only avoiding manual feature extraction, but they can also automatically extract high-dimensional features that are difficult to understand and discover, and they can also classify and predict data automatically. They are a leading approach to a variety of computer vision problems because of their many advantages. With the emergence of well-known successors like AlexNet^25^, VGGNet^26^, and GoogleLeNe^27^, the development of CNNs has been significantly modified in terms of layering and block design. Deep learning models all share the ability to learn high-dimensional representations from large datasets regardless of the network structure. This section provides a detailed overview of related research on deep learning methods.

Zhao et al.^28^ merged four micro-expression data sets into a composite micro-expression dataset, then used Eulerian video amplification (EVM) technique to amplify facial actions and extract optical flow features, and finally designed a shallow CNN network to extract features and classify micro-expression classes. Peng et al.^29^ used large-size The ResNet10 network was pre-trained on the ImageNet dataset using emoji data and later fine-tuned on the micro-expression dataset, with higher metrics than the base methods such as LBP-TOP and HOOF on both composite datasets. The same authors^30^, first used a medium-sized end-to-end neural network model, namely a dual time-scale convolutional neural network (DTSCNN), to recognize micro-expressions. The model has two temporal channels and is designed for data with different temporal properties. For example, the cameras used to collect the data have different frame rates. Only four convolutional layers and four pooling layers are used in each channel to avoid overfitting. The achieved recognition rate is about 10% higher than some previous state-of-the-art methods. Huai-Qian et al.^31^ proposed to train a network with convolutional and recursive layers for micro-expression recognition. Instead of using data enhancement on the dataset, they extracted optical flow features to enrich the input for each time step or specific time length. However, they did not achieve competitive results due to the occurrence of overfitting situations caused by using deep networks on small datasets. Moreover, for the nature of micro-expressions with durations less than 1/2 s, training the network on complete video clips may not be appropriate. Since the sample size of micro-expression datasets is still small, they cannot be adequately trained using deep convolutional neural networks, and the training cost is high, making it difficult for ordinary computer hardware resources to meet the training requirements. Xia et al.^32^ analyzed composite datasets and concluded that low-resolution and shallow network models can help improve the accuracy of models trained on composite datasets, and proposed a recursive convolutional network with partially parameter-free module of a recursive convolutional network to validate their arguments, and the results showed that the proposed method outperformed the state-of-the-art methods. Another study by this author^33^ proposed an end-to-end framework consisting of a recursive convolutional network (RCNN) to recognize micro-expressions. The RCNN was used to learn subtly varying features and recognize micro-expressions. Song et al.^21^ proposed a three-stream convolutional neural network (TSCNN) for micro-expression recognition and designed a TSCNN module for dynamic temporal flow, static spatial flow, and local spatial streams to learn and integrate temporal, whole face region and local face region cues from micro-expression videos for micro-expression recognition, respectively. The results are also compared with many state-of-the-art methods in research. Temporal dithering was used to enrich the training samples to facilitate the learning process, and the effectiveness of the method was validated on three spontaneous micro-expression datasets.

In recent studies, researchers have also tried to design or use lightweight neural networks to reduce the memory resources and computational resources occupied during the training process. Belaiche et al.^34^ proposed an optimized network based on ResNet18 to run faster and reduce the memory footprint while reducing the network depth. In addition, a more compact optical flow feature representation is used, which allows the network to be more time-efficient while having more substantial accuracy. Liu et al.^35^ proposed an improved MobileViT model that combines a convolutional neural network and a lightweight ViT module to improve the accuracy of the lightweight network in recognizing micro-expressions without increasing the training cost. Xu et al.^36^ used the equally efficient and lightweight MobileNetV2 and made a significant improvement in the classification accuracy of their network model by adjusting the classifier. They also obtained more beneficial information for recognition by extracting the optical flow features of the samples.

By adapting the training approaches and improving the lightweight neural network, the model can be trained in the same or shorter time and the results are not significantly different from those of the state-of-the-art.

## Micro-expression recognition model design

Following is a description of the proposed model.

1. Preprocessing: facial alignment and cropping of micro-expression image sequences, from which onset and apex frames are later derived.
2. The TV-L1 optical flow feature extraction algorithm extracts the light change features between the apex frame and the onset frame as a result of muscle movement.
3. Pre-training of TV-L1 optical flow features with an improved ShuffleNet network: The extracted TV-L1 optical flow features are fed into the network model separately for pre-training and saving the weights when accuracy is high. Pre-training makes it easier for the network model to detect subtle facial movements.

## Classification of pre-processed image sequences using improved ShuffleNet network: load the model weights from pre-training and classify the image sequences.

**Figure 1:**
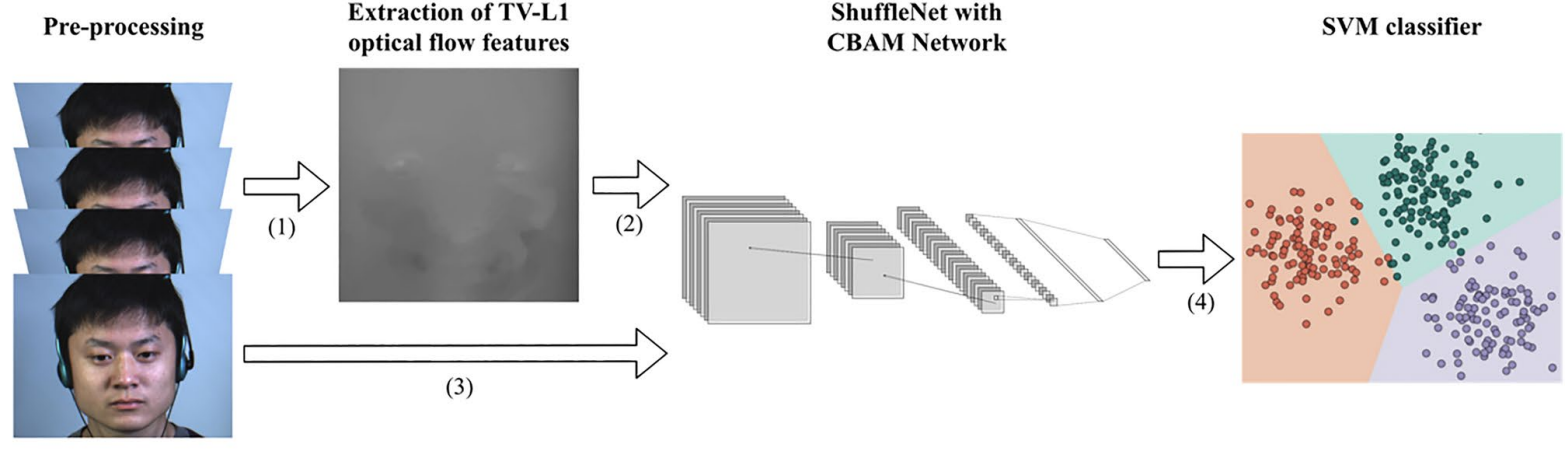
Micro-expression recognition model. (1) Obtain the start frame and vertex frame from the pre-processed sequence and extract the TV-L1 optical flow features between them. (2) Feed the optical flow features into the improved ShuffleNetV2 for pre-training and get the weights. (3) Load the weights and feed the pre-processed dataset into the improved ShuffleNetV2. (4) Use the SVM classifier in training for classification. Images are from subject 1 of the CASME II dataset^6^ and is reproduced here following the CASME2 license agreement. Copyright © Xiaolan Fu.

Figure 1 illustrates the simplified flow chart of this model.

### Pre-processing

**Figure 2:**
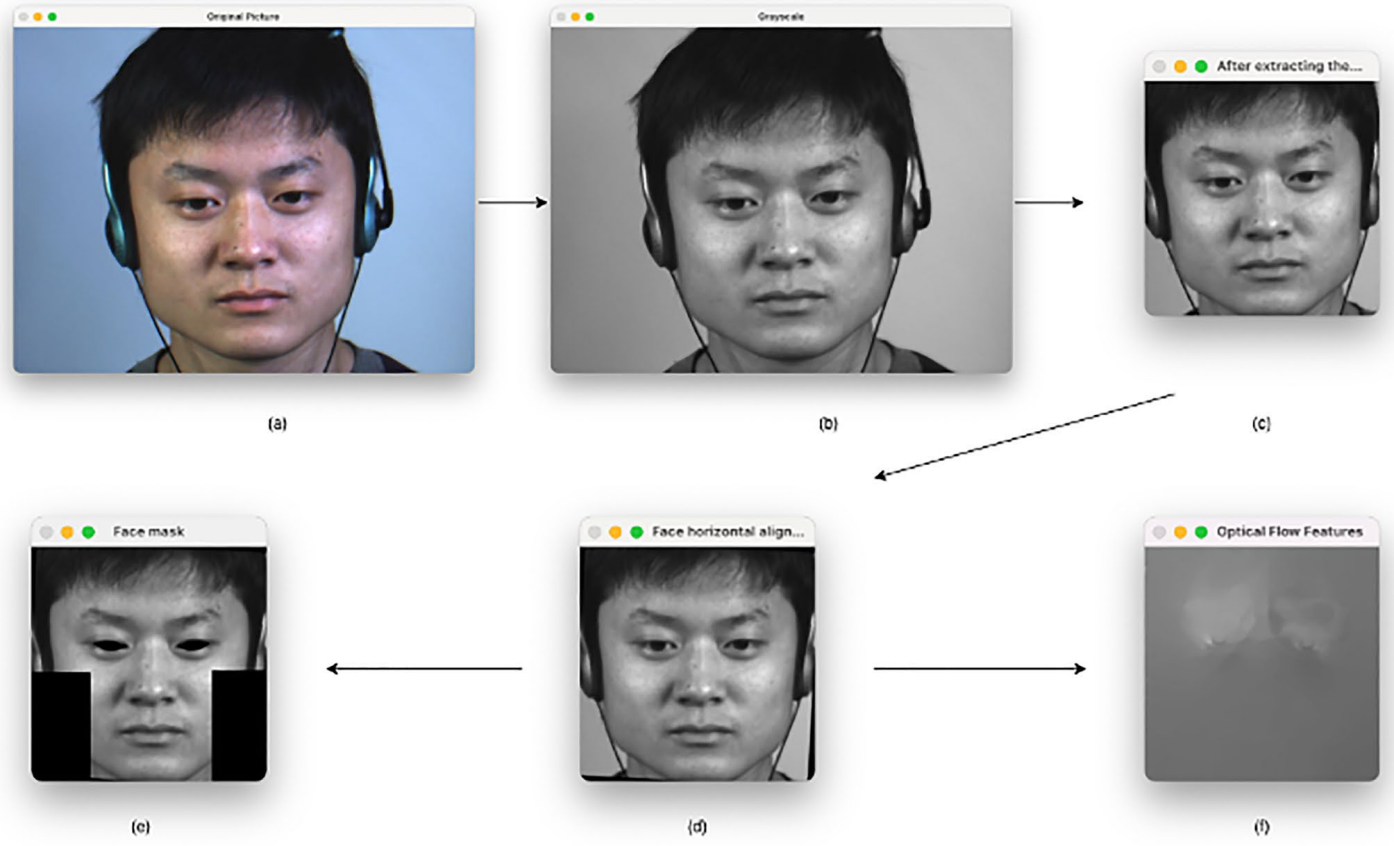
(**a**) Raw image. (**b**) Grayscale processing of the raw image. (**c**) Extracting the face and cropping the size. (**d**) Rotating the face to horizontal. (**e**) Adding facial masks. (**f**) Extracting optical flow features. Images are from subject 1 of the CASME II dataset^6^ and is reproduced here following the CASME2 license agreement. Copyright © Xiaolan Fu.

The sampling equipment and sampling conditions used to build the dataset can also vary from institution to institution, which can result in large differences in the data resolution, the code rate, and other metrics in the dataset. The data from different datasets are processed equally in this study, and the schematic diagram of the pre-processing steps is shown in Fig. 2. We utilize the original expression image sequences provided by the dataset for processing.

After obtaining the complete microexpression image sequence, we use the OpenCV and Dlib toolkit to extract the face, position the facial marker points, and crop the face to the size of $224\times 224$ pixels. The face then needs to be aligned to ensure that it is always horizontal. The 68 facial landmark points detector provided by the Dlib toolkit was used to obtain the coordinates of the left eye $A{(x}_{L},{y}_{L})$ and the right eye ${B(x}_{R},{y}_{R})$ and calculate the coordinates $C({x}_{C},{y}_{C})$ of the center of the two eyes as the center of rotation. The angle $\gamma$ of the desired rotation is then obtained by calculating the tangent of the horizontal and vertical distances between the left eye and the center point, and the face picture is rotated to horizontal by the affine transformation, and the angle is calculated as in Eq. 1.

1 $$\gamma =\mathit{arctan}\frac{{y}_{C}{- y}_{A}}{{x}_{C}-{x}_{A}},$$

After rotation to horizontal, optical flow features are extracted between the onset and apex frames. As a final step, eye regions and regions that produce little or no movement are masked by marked facial marker points so as to avoid noise caused by eye movements (e.g., blinking) and to reduce redundant features. The micro-expression dataset did not contain sufficient data, and the number of expressions in different categories was extremely unbalanced after counting the samples, which could easily result in bias in the neural network. The positive emotion as well as the surprise emotion were therefore expanded by horizontal inversion. The number of emotion categories has been essentially balanced after the expansion.

### TV-L1 optical flow features and pre-training

The movements of micro-expressions are very subtle, lasting less than 0.2 s. In addition, all samples used in this study were collected in a laboratory under fixed conditions. Consequently, each micro-expression sample satisfies the conditions for using optical flow features, which are that the luminance and channel values remain essentially constant, and that pixels move very little between adjacent frames.

Suppose the light energy of a point in the first frame is denoted as $f( x, y, t )$, where $( x , y )$ is the pixel coordinate and $t$ is the time, after time $dt$, the point has moved $dx, dy$ distance. The intensity of the two adjacent frames is achieved as:

2 $$f\left(x,y,t\right)=I\left(x+dx,y+dy,t+dt\right),$$

From the study of Xu et al.^36^ by performing a Taylor expansion of Eq. (2), it is obtained that:

3 $$f\left(x,y,t\right)=f\left(x,y,t\right)+\frac{\partial f}{\partial x}dx+\frac{\partial f}{\partial y}dy+\frac{\partial f}{\partial t}dt,$$

4 $$\frac{\partial f}{\partial x}dx+\frac{\partial f}{\partial y}dy+\frac{\partial f}{\partial t}dt=0,$$

Dividing both sides of the equation equally by dt yields:

5 $${f}_{x}u+{f}_{y}v+{f}_{t}=0,$$

6 $${f}_{x}=\frac{\partial f}{\partial x};{f}_{y}=\frac{\partial f}{\partial y}dy,$$

7 $$u=\frac{dx}{dt};v=\frac{dy}{dt},$$

where $u$ and $v$ are the horizontal and vertical components of the optical flow field, respectively. But assuming a constant brightness for 1 will only produce a constraint equation that is not sufficient to solve for u and $v$. Therefore, solving the optical flow field model requires an additional constraint on the displacement field vector. It has been proposed to add a smoothing constraint to the optical flow constraint, which assumes that the velocity of the object motion is locally smooth in most cases^37^. In particular, when the target is in rigid motion without deformation, the velocity of each neighboring pixel point should be the same, i.e., the rate of change of the velocity of the neighboring points is zero, and the solution of the optical flow field is transformed into the energy generalized minimal value problem:

8 $$\underset{u,v}{min} \left\{{E}_{\mathrm{H}-\mathrm{S}}={\int }_{\Omega } \left(|\nabla u{|}^{2}+|\nabla v{|}^{2}\right)\mathrm{d\Omega }+\lambda {\int }_{\Omega } {\left({f}_{x}u+{f}_{y}v+{f}_{t}\right)}^{2}\mathrm{d\Omega }\right\},$$

The first term is a regular term that assumes that the optical flow field does not show large variations and smooth velocity fields can be obtained. The second term is the data term, the basic optical flow constraint, which assumes constant grayscale values before and after the corresponding point motion. $\lambda$ is the regularization parameter, which is a weighted parameter associated with the regular term and the data term. Since the regular term of H–S model adopts smoothness constraint and both the regular term and data term are quadratic, the robustness is poor and cannot keep the discontinuity of displacement field, which will cause serious blurring and loss of important information during the image evolution. To overcome the shortcomings of the H–S model, a TV-L1 optical flow model based on the full variational approach is proposed^38^ and the optical flow constraint is improved by introducing the variable *w*. Modeling of $w$ against light changes is obtained:

9 $$\underset{u,v,w}{min} \left\{{E}_{\mathrm{TV}-\mathrm{L}1}={\int }_{\Omega } \left(\left|\nabla u\right|+\left|\nabla v\right|+\left|\nabla w\right|\right)d\Omega +\lambda {\int }_{\Omega } \mid \rho \left(u,v,w\right)d\Omega \right\},$$

where $\rho (u,v,w)={I}_{x}\left(u-{u}_{0}\right)+{I}_{y}\left(v-{v}_{0}\right)+{I}_{t}+\beta w$. The parameter $\beta$ is a factor for the weight light change term. Although there is little change compared with the H–S model, the alignment accuracy has been greatly improved. First, the total variation regular term maintains the discontinuity of the displacement field and protects the edge information from being blurred during the diffusion. The replaced regular term has a good denoising effect while keeping the edge information from being blurred. Secondly, the data items of TV-L1 are less sensitive to luminance changes compared to the H–S model. In the experiments, the more robust TV-L1 approach was chosen.

First of all, as part of the pre-training, we extracted the same number of samples from each category in order to prevent the network model from being biased towards a specific category of emotions during classification. After that, the feature images are fed into the network model randomly without repetition and made to perform classification. In this process, the initial learning rate is set to 0.001, the cross-entropy loss function is used to calculate the loss, and the Softmax classifier is used to classify the images. In pre-training, a final accuracy of 96.28% was achieved for identifying optical flow feature images and model weights were saved.

### ShuffleNet model combined with CBAM

Self-attention-based ViT methods have achieved excellent results in many computer vision problems over the past few years, however their models tend to be very complex. A pair of approaches is adopted in this paper in order to optimize the accuracy of micro-expression recognition and maintain the lightweightness of the models. To form a new network model, a convolutional block attention module is added to ShuffleNetV2 as the first approach. As a result of this approach, attention maps can be added to feature maps in any CNN model structure at a non-negligible time–space overhead. In addition, incorporating the improved lightweight transformer module with ShuffleNetV2 is an alternative approach, as it contains the full operation of the transformer and retains CNN-specific features such as inductive bias and global nature of the transformer. The overall model can still remain lightweight and accurate by improving the transformer block.

#### Convolutional block attention module

A lightweight convolutional block attention module for feedforward neural networks has been proposed in 2018, which can infer the attentional feature map from two independent dimensions, channel and space, and then multiply the attentional feature map with the input feature map to refine the features adaptively. In view of its generality, the module can be seamlessly integrated into any CNN architecture with minimal time and space overhead.

The spatial information of the feature map is first aggregated by using the average pooling and maximum pooling operations to generate two different spatial context descriptors: ${{\varvec{F}}}_{{\varvec{a}}{\varvec{v}}{\varvec{g}}}^{{\varvec{c}}}$ and ${{\varvec{F}}}_{{\varvec{m}}{\varvec{a}}{\varvec{x}}}^{{\varvec{c}}}$, which denote the average pooling feature and the maximum pooling feature respectively. The two descriptors are then forwarded to the shared network to generate the channel attention map ${\mathbf{M}}_{\mathbf{c}}\in {\mathbb{R}}^{C\times 1\times 1}$. The shared network consists of a multilayer perceptron (MLP) with one hidden layer. To reduce the parameter overhead, the hidden activation size is set to ${\mathbb{R}}^{C/r\times 1\times 1}$, where $r$ is the scaling rate. After applying the shared network to each descriptor, we merge the output feature vectors using element-by-element summation. In brief, channel attention is calculated as follows:

10 $$\begin{array}{c}{\mathbf{M}}_{\mathbf{c}}\left(\mathbf{F}\right)=\sigma \left(MLP\left(\mathrm{AvgPool}\left(\mathbf{F}\right)\right)+MLP\left(\mathrm{MaxPool}({\varvec{F}})\right)\right)\\ =\sigma \left({\mathbf{W}}_{1}\left({\mathbf{W}}_{0}\left({\mathbf{F}}_{\mathbf{a}\mathbf{v}\mathbf{g}}^{\mathbf{c}}\right)\right)+{\mathbf{W}}_{1}\left({\mathbf{W}}_{0}\left({\mathbf{F}}_{\mathbf{m}\mathbf{a}\mathbf{x}}^{\mathbf{c}}\right)\right)\right)\end{array},$$

where $\sigma$ denotes the sigmoid function, ${\mathbf{W}}_{0}\in {\mathbb{R}}^{C/r\times C}, {\text{and}}{\mathbf{W}}_{1}\in {\mathbb{R}}^{C\times C/r}$. Note that the two inputs share the MLP weights ${\mathbf{W}}_{0}$ and ${\mathbf{W}}_{1}$, and ${\mathbf{W}}_{0}$ followed by the ReLU activation function.

Spatial attention maps are generated by exploiting the spatial interrelationships of features. Unlike channel attention, spatial attention is focused on "where" as an information component that complements channel attention. To compute spatial attention, first apply the average pooling and maximum pooling operations along the channel axes and concatenate them to generate a valid feature descriptor. Applying pooling operations along the channel axis is effective in highlighting areas of information. On the connected feature descriptors, a convolutional layer is applied to generate a spatial attention map ${\mathbf{M}}_{\mathbf{s}}(\mathbf{F})\in {\mathbf{R}}^{H\times W}$, which encodes the emphasized or suppressed locations.

**Figure 3:**
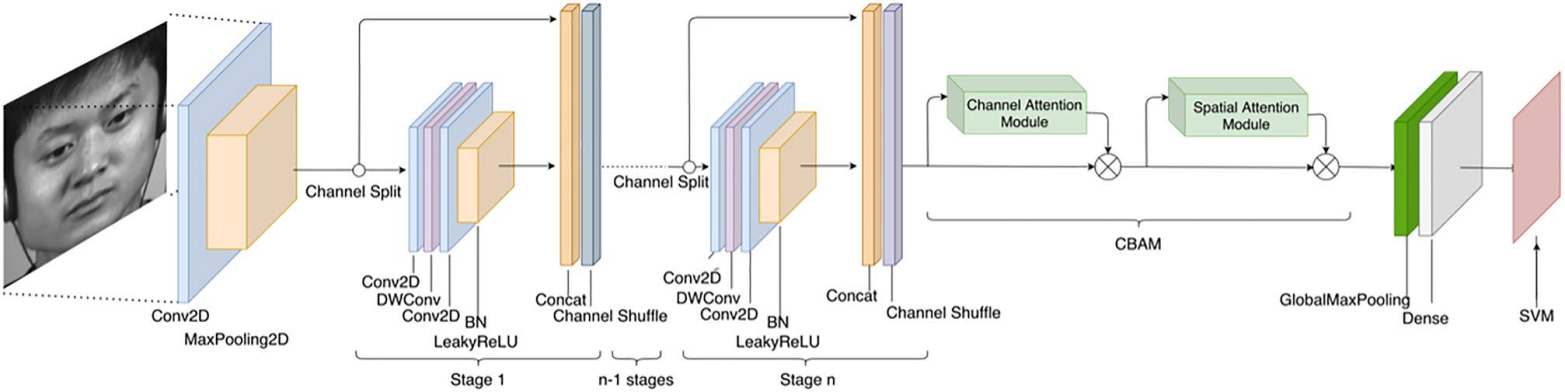
ShuffleNet network structure diagram combined with CBAM. Images are from subject 1 of the CASME II dataset^6^ and is reproduced here following the CASME2 license agreement. Copyright © Xiaolan Fu.

Two two-dimensional maps are generated by using two pooling operations to aggregate the channel information of the feature maps: ${\mathbf{F}}_{\mathbf{a}\mathbf{v}\mathbf{g}}^{\mathbf{s}}\in {\mathbb{R}}^{1\times H\times W}$ and ${\mathbf{F}}_{\mathbf{m}\mathbf{a}\mathbf{x}}^{\mathbf{s}}\in {\mathbb{R}}^{1\times H\times W}$. Denotes the average pooling feature and the maximum pooling feature for the whole channel. These are connected and convolved by a standard convolutional layer to generate a 2D spatial attention map. In brief, spatial attention is calculated as follows:

11 $$\begin{array}{c}{\mathbf{M}}_{\mathbf{s}}\left(\mathbf{F}\right)=\sigma \left({f}^{7\times 7}\left(\left[\mathrm{AvgPool}\left(\mathbf{F}\right);\mathrm{MaxPool}\left(\mathbf{F}\right)\right]\right)\right)\\ =\sigma \left({f}^{7\times 7}\left(\left[{\mathbf{F}}_{\mathbf{a}\mathbf{v}\mathbf{g}}^{\mathbf{s}};{\mathbf{F}}_{max}^{\mathbf{s}}\right]\right)\right)\end{array}$$

where $\sigma$ denotes the sigmoid function and ${f}^{7\times 7}$ denotes the convolution operation with a filter size of $7\times 7$. The complete model structure is shown in Fig. 3.

#### Improved lightweight ViT module

Various tasks, including image classification, target detection, and semantic segmentation, are significantly improved by the original ViT over CNNs. Nevertheless, these performance improvements usually require high computational resources. For example, DeiT^39^ multi-adds more than 10 G of computational resources to perform image classification tasks. These high computational resource requirements are beyond the capabilities of many devices. It is also difficult to capture subtle facial changes with lightweight and efficient convolutional neural networks designed for mobile vision tasks due to localization problems in extracting features. As a result, we hope to combine ViT with ShuffleNetV2 to complement one another.

Our improvements for the ViT module mainly include the way in which the self-attention is computed and the different image encoding methods ensure that the module contains local feature extraction capabilities similar to convolutional layers and speeds up the training efficiency of the ViT module. First, we note that the underlying transformer consists of alternating multi-headed self-attention and multilayer perceptron (MLP), while the calculation of self-attention can be expressed by Eq.

12 $$\mathrm{Self}-\mathrm{Attention}\left(\mathrm{Q},\mathrm{K},\mathrm{V}\right)=\mathrm{Softmax}\left(\frac{Q{K}^{T}}{\sqrt{{d}_{k}}}\right)V$$

where $Q, K, V$ are query, key and value matrices, dk is the query/key channel dimension, $N$ is the number of tokens, and $C$ is the token channel dimension. In a lightweight model with limited capacity, the computational cost of self-attentiveness is higher than that of convolutional layers. The computational complexity of self-attention is quadratic with respect to the spatial resolution and introduces three linear layers of the same level to compute linear combinatorial results of $V$. To alleviate this problem, we received inspiration to introduce the Ghost module to replace the linear layers in self-attention, which uses ordinary convolution to generate some inherent feature maps, and then enhances the features and adds channels using linear operations with less computational overhead to obtain better performance and speed performance. Secondly, the weight sharing mechanism is utilized to reuse the weights in $Q$, $K$, and $V$ calculations and to reuse the features $V$ into $Q$ and $K$. This is due to the fact that Ma et al.^40^ argue that $Q$ and $K$ are only involved in the computation of the attentional map, while the final result of the self-attention mechanism is a linear combination of each token in $V$. Compared with $Q$ and $K$, $V$ needs to retain more semantic information to ensure the final weighting and the representational power of the result. Thus the results of the self-attentive mechanism are strongly correlated with $V$ but weakly correlated with $Q$ and $K$. Therefore simplifying the computation of $Q$ and $K$ can lighten the overall computational overhead of the model more. The weight sharing mechanism can be expressed as follows:

13 $$\begin{array}{c}{f}^{v}={f}^{k}={f}^{q}\\ V={f}^{v}\left(X\right)\\ K={f}^{k}\left(X\right)=\mathrm{Identity}\left({V}^{T}\right)\\ Q={f}^{q}\left(X\right)=\mathrm{Identity}\left(V\right)\end{array}$$

where ${f}^{v},{f}^{k}\text{ and }{f}^{q}$ are the projections for calculating $Q$, $K$, and $V$, respectively. The improved calculation of self-attentiveness can be written as:

14 $${\text{new Self-Attentiveness}}\left(V\right)=\mathrm{Softmax}\left(\frac{V{V}^{T}}{\sqrt{{d}_{v}}}\right)V$$

Finally, before feeding the feature map into the transformer module, an $n\times n$ standard convolutional layer is first applied to the feature map, after which the features are generated using point convolution. $n\times n$ convolutional layers encode local spatial information, and point convolution projects the tensor into high-dimensional space by learning linear combinations of the input channels. First, a standard convolutional layer with $n\times n$ is applied for a given image input tensor ${\varvec{X}}\in {\mathbb{R}}^{H\times W\times C}$ (where $H, W, C$ are the width, height and number of channels of the image, respectively), to which a point convolutional layer is connected, yielding ${\mathbf{X}}_{L}\in {\mathbb{R}}^{H\times W\times d}$. $n\times n$ convolutional layer encodes local spatial information. In contrast, the point convolutional layer projects the tensor to the high-dimensional space by learning linear combinations of the input channels. The method is to expand ${\mathbf{X}}_{L}$ into N non-overlapping flattened blocks ${\mathbf{X}}_{U}\in {\mathbb{R}}^{P\times N\times d}$ where $P=wh$, $N=\frac{HW}{P}$ is the number of blocks, and $h\le n$ and $w\le n$ are the height and width of a block, respectively. For each $p\in \{1,\cdots , P\}$, the relationship between the blocks is encoded by applying our improved transformer. The ${\mathbf{X}}_{G}\in {\mathbb{R}}^{P\times N\times d}$ was obtained:

15 $${\mathbf{X}}_{{\varvec{G}}}\left({\varvec{p}}\right)=\mathbf{T}\mathbf{r}\mathbf{a}\mathbf{n}\mathbf{s}\mathbf{f}\mathbf{o}\mathbf{r}\mathbf{m}\mathbf{e}\mathbf{r}\left({\mathbf{X}}_{{\varvec{U}}}\left({\varvec{p}}\right)\right),1\le {\varvec{p}}\le {\varvec{P}}$$

**Figure 4:**
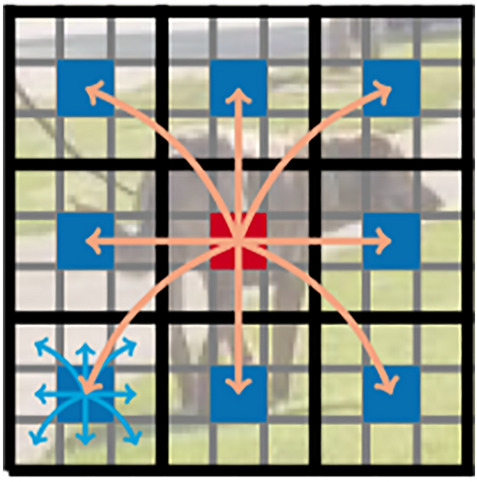
Each pixel sees every other pixel in our ViT block. In this example, the red pixel uses the transformer to focus on the blue pixel (the pixel in the corresponding position in the other blocks). Because the blue pixel already encodes the information of neighboring pixels using convolution, this allows the red pixel to encode the information of all pixels in the image. Here, each cell in the black and grey grid represents a block and a pixel, respectively^41^.

**Figure 5:**
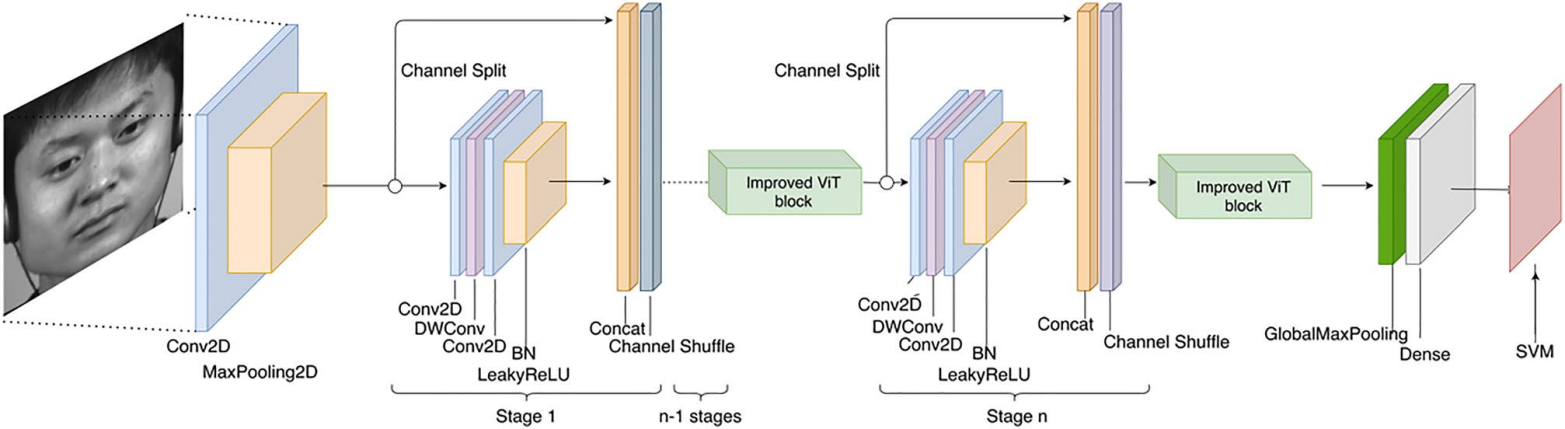
In this model, the convolutional layer of ShuffleNetV2 is responsible for downsampling, and a modified ViT module is inserted before and after the last ShuffleNetV2 layer to extract global features.

Unlike vanilla ViT, which loses the spatial order of pixels, our ViT blocks loses neither the block order nor the spatial order of pixels within each block. Therefore, ${\mathbf{X}}_{G}\in {\mathbb{R}}^{P\times N\times d}$ can be collapsed to obtain ${\mathbf{X}}_{F}\in {\mathbb{R}}^{H\times W\times d}$. Then, ${\mathbf{X}}_{F}$ is projected to the low C-dimensional space using point-to-point convolution and combined with ${\varvec{X}}$ by the join operation. Another $n\times n$ convolution layer is then used to fuse the local and global features in the tandem tensor and output. Since ${\mathbf{X}}_{U}(p)$ uses convolution to encode local information in the n × n region and ${\mathbf{X}}_{G}(p)$ encodes the global information of the P blocks at the pth position, each pixel in ${\mathbf{X}}_{G}$ can encode the information of all pixels in ${\varvec{X}}$, as shown in Fig. 4. Thus, our ViT block has no loss of an effective sensory field. The model structure after the introduction of the improved ViT module is shown in Fig. 5.

#### ShuffleNetV2

ShuffleNetV2 is an upgraded version proposed by Face + + and Tsinghua University in 2018 for ShuffleNetV1, which is more accurate than ShuffleNetV1 and MobileNetV2 with the same complexity. By analyzing ShuffleNetV1, the researchers found that point-by-point group convolution and bottleneck structure increase the memory access cost(MAC) and the cost is not negligible. Element-level addition operations in shortcut connections will also result in reduced efficiency.

**Figure 6:**
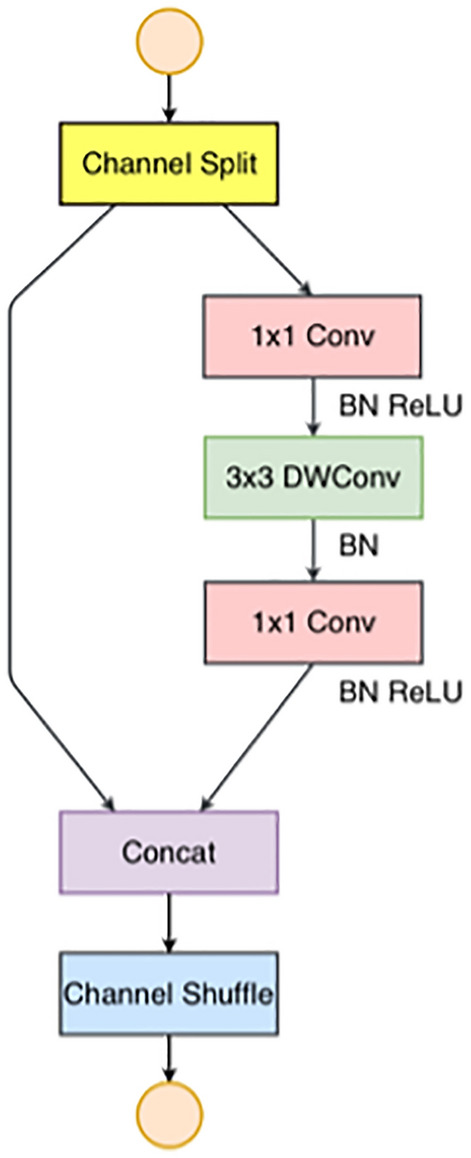
ShuffleNetV2 channel splitting schematic.

ShuffleNetV2 introduces channel splitting, as shown in Fig. 6, by dividing the input feature channels into two branches, one of which remains unchanged and the other consists of three convolutions with the same input and output channels, and two of the $1\times 1$ convolutions are no longer group convolutions, ensuring minimal memory access cost.

By doing the above, the modules in the ShuffleNetV2 network can use more channel features and larger network capacity, thus maintaining high accuracy and reducing computational complexity. In addition, due to channel segmentation, part of the features can be connected to the following blocks directly, which is equivalent to feature reuse, making the network not only efficient but also accurate. The network structure used in this paper is shown in Table 1.

**Table 1:** ShuffleNetV2 network structure.

Layers	Output size	Kernel Size	Stride	Repeat	Output channels
Image	224 × 224	–	–	–	3
Conv1	112 × 112	3 × 3	2	1	24
MaxPool	56 × 56	3 × 3	2		
Stage 2	28 × 28	1 × 1	2	1	116
	28 × 28	1 × 1	1	3	
Stage 3	14 × 14	1 × 1	2	1	232
	14 × 14	1 × 1	1	7	
Stage 4	7 × 7	1 × 1	2	1	464
	7 × 7	1 × 1	1	3	
Conv 5	7 × 7	1 × 1	1	1	1024
GlobalPool	1 × 1	7 × 7	–	–	–

Basic ShuffleNetV2 uses the SoftMax classifier. SoftMax regression is an improvement based on logistic regression for multi-classification problems, and when a given sample is input, it outputs a value between 0 and 1, indicating the probability that the input sample belongs to that class. For the expression image ${\varvec{x}}$, the probability of its class j is:

16 $$p\left({y}^{\left(i\right)}=j\mid {\mathbf{x}}^{\left(i\right)},\theta \right)=\frac{{e}^{{\theta }_{J}^{T}{\mathbf{x}}^{\left(i\right)}}}{\sum_{i=1}^{k} {e}^{{\theta }_{J}^{T}{\mathbf{x}}^{\left(i\right)}}},$$

where $p\left({y}^{(i)}=j\mid {\mathbf{x}}^{(i)},\theta \right)$ is the probability that the expression image ${\varvec{x}}$ corresponds to each expression category $j$, and $\theta$ is the parameter to be fitted. The category with the highest probability value is the final result of the neural network prediction classification. Because the number of samples in the micro-expression dataset is small and the differences between different emotion samples of the same subject are very subtle. Since the SoftMax classifier minimizes the cross-entropy from a global perspective, the SoftMax classifier will likely lead to misclassification once there are samples with large differences. To solve this problem, and because SVM performs well on small data sets and often has higher classification accuracy than other classifiers. We also try to use the SVM classifier for classification. SVM tries to find the maximum margin between data points of different categories. It has better differentiability, and the regularization term penalizes the wrongly scored data more strongly, with strong generalization capabilities, thus facilitating the differentiation of micro-expression features. Its Hinge loss function is defined as:

17 $$\mathrm{loss}\left(\mathbf{x},\mathrm{y}\right)=\frac{1}{N}\sum_{i=1,i\ne y}^{N} max\left(0,{\left(\mathrm{margin}-{\mathbf{x}}_{y}+{\mathbf{x}}_{i}\right)}^{p}\right),$$

where 1 ≤ y ≤ N denotes the label.

In the experiments L2 regularization was added to the model, by adding regularization it allows the model to use as many features as possible to identify emotional features rather than individual features. Besides, replacing the ReLU activation function with LeakyReLU can avoid the occurrence of neuron death effectively.

## Experiments

### Dataset

In this paper, four representative datasets are selected for training and testing the proposed approach: the SMIC^7^, CASME II^6^, SAMM^8^ and MMEW^9^, and the three selected datasets were all specifically designed for the detection and recognition of spontaneous micro-expressions. The SMIC dataset contained 164 micro-expression segments from 16 subjects, six females and ten males. The collection is done by cameras with different frame rates. These subjects underwent emotion capture in an interrogation room setting, and the experiment also contained punishment and threat mechanisms that suppressed irrelevant facial expressions of the subjects. The CASMEII dataset contains 247 micro-expression clips from 26 subjects, and the camera equipment used for acquisition has a frame rate of 200 fps, allowing for more images to be produced in the same amount of time compared to SMIC, and a greater increase in image resolution. The SAMM dataset contains 159 micro-expression segments from 32 subjects, achieving gender balance while including multiple ethnicities and nationalities, with a resolution of $400\times 400$ in the facial region, the highest of any dataset to date. In addition, the acquisition process uses a variety of LED devices to ensure stable light. The newly released MMEW dataset uses the same size resolution of facial regions as SAMM, and the overall image resolution is second only to SAMM. MMEW contains 300 micro-expression samples from 36 subjects with an average age of 22.35 years, the largest sample size of any micro-expression dataset. However, its shortcoming is that the frame rate is only 90, which is lower than the other three datasets.

All of the above datasets are publicly available, and each dataset has been approved for use by the relevant authorities. Our experimental protocols and the images in the text are also approved for use.

To keep the classification consistent across the three datasets, the expression types in MMEW, CASMEII and SAMM were combined into three categories in the experiment. Among them, happy is obviously a positive emotion, so it is classified as a positive emotion. On the contrary, disgust, sadness, fear, anger, and contempt usually represent negative emotions of people, so these emotions are classified as negative emotions. And surprise cannot be judged to be caused by good or bad things directly, so such expressions need further judgment.

### Experiment settings and performance evaluations

In the experiment, the deep learning part uses the Keras framework to build the neural network structure, the hardware platform CPU is Intel Core i99980HK, the memory is 32 GB, and the graphics card model is AMD Radeon Pro 5500 M. Evaluation was performed on a composite dataset consisting of a combination of the most commonly used CASMEII, SAMM, and SMIC datasets. Similarly, this experiment also applied the leave-one-out cross-validation approach for micro-expression recognition classification, where one subject's data was used as the test set in each iteration of the cross-validation. The advantage of using this approach is that it can better reproduce the situation when new subjects are encountered during model training and improves the generalization ability of the model. This way of evaluating the generalization ability of the model is gradually becoming more mainstream in micro-expression recognition tasks. The Adam optimizer is used to train the model with an initial learning rate of $10e-3$, and the learning rate is dynamically adjusted by accuracy and loss. In order to make a fair comparison with other studies, we use the unweighted recall rate (UAR) and the unweighted F1 score (UF1) as evaluation metrics alongside the accuracy rate. These metrics allow the accuracy of model recognition and the ability to balance between different classes to be measured. Assume that ${\varvec{T}}{\varvec{P}}$, ${\varvec{F}}{\varvec{P}}$ and ${\varvec{F}}{\varvec{N}}$ are the true positive, false positive and false negative, respectively. The UAR is calculated by ${\varvec{U}}{\varvec{A}}{\varvec{R}}=\frac{1}{{\varvec{C}}}\sum_{{\varvec{c}}=1}^{{\varvec{C}}} \frac{{\varvec{T}}{{\varvec{P}}}_{{\varvec{c}}}}{{{\varvec{N}}}_{{\varvec{c}}}}$ where ${\varvec{T}}{{\varvec{P}}}_{{\varvec{c}}}$ and ${{\varvec{N}}}_{{\varvec{c}}}$ are the number of true positives and all samples in ${\varvec{c}}$-th class. The UF1 is computed as ${\varvec{U}}{\varvec{F}}1=\frac{1}{{\varvec{C}}}\sum_{{\varvec{i}}={\varvec{c}}}^{{\varvec{C}}} \frac{2{{\varvec{P}}}_{{\varvec{c}}}\times {{\varvec{R}}}_{{\varvec{c}}}}{{{\varvec{P}}}_{{\varvec{c}}}+{{\varvec{R}}}_{{\varvec{c}}}}$, where ${{\varvec{P}}}_{{\varvec{c}}}=\frac{{\varvec{T}}{{\varvec{P}}}_{{\varvec{c}}}}{{\varvec{T}}{{\varvec{P}}}_{{\varvec{c}}}+{\varvec{F}}{{\varvec{P}}}_{{\varvec{c}}}}$ and ${{\varvec{R}}}_{{\varvec{c}}}=\frac{{\varvec{T}}{{\varvec{P}}}_{{\varvec{c}}}}{{\varvec{T}}{{\varvec{P}}}_{{\varvec{c}}}+{\varvec{F}}{{\varvec{N}}}_{{\varvec{i}}}}$ for ${\varvec{c}}$-th class.

The convolutional and fully-connected layers of the model were subjected to L2 regularization at a scale of 0.2 and Dropout at a scale of 0.5 to avoid overfitting. To improve the speed of each part of the experiment, the Keras 2.2.4 library was utilized.

### Results and discussion

In the experiments to improve the accuracy we added pre-training of optical flow features and models composed of different modules, so to prove the effectiveness of these approaches, ablation experiments are essential. Table 2 shows the effect of the different methods on the performance metrics. Firstly, for whether the pre-training of TV-L1 optical flow features is effective for micro-expression recognition, we set up two sets of experiments, one without pre-training optical flow features and the other with pre-training and loading the weights obtained from pre-training, and in the experiments, we used different network models to verify the robustness of the methods. These include lightweight convolutional neural networks and regular convolutional neural networks, and a pure ViT model without convolutional structure is also added. The experimental results show that this TV-L1 optical flow feature pre-training can make it easier for ShuffleNetV2 to observe the subtle changes of human faces in formal training, proving that the TV-L1 optical flow feature can effectively reflect the characteristics of micro-expressions.

**Table 2:** Ablation experiments to verify the effect of different methods and modules on model recognition accuracy.

Approach	MMEW		CASME II		SMIC		SAMM	
	UAR	UF1	UAR	UF1	UAR	UF1	UAR	UF1
ShuffleNetV2 without pre-trained	0.6252	0.6384	0.6336	0.6497	0.6180	0.6104	0.6149	0.6214
ShuffleNetV2 without SVM classifier	0.6222	0.6392	0.6207	0.6291	0.6038	0.5918	0.5925	0.5899
ShuffleNetV2	0.6524	0.6621	0.6647	0.6583	0.6394	0.6451	0.6712	0.6592
ShuffleNetV2 with CBAM	0.6684	0.6719	0.6739	0.6619	0.6581	0.6610	0.6802	0.6694
ShuffleNetV2 with improved ViT	**0.6894**	**0.7251**	**0.6925**	**0.7008**	**0.7159**	**0.7141**	**0.6684**	**0.7410**
MobileNetV2 without pre-trained	0.5882	0.5741	0.5741	0.5726	0.6150	0.6021	0.5632	0.6131
MobileNetV2 without SVM classifier	0.6091	0.6032	0.5744	0.5951	0.5886	0.6125	0.5869	0.6262
MobileNetV2^42^	0.6448	0.6684	0.6358	0.6229	0.6768	0.6692	0.6328	0.6517
ResNet50 without pre-trained	0.6427	0.6231	0.6364	0.6253	0.6331	0.6480	0.5905	0.6059
ResNet50 without SVM classifier	0.6438	0.6571	0.6296	0.6308	0.6018	0.6319	0.6246	0.6581
ResNet50	0.6793	0.6782	0.6743	0.6682	0.6841	0.6621	0.6599	0.6827
DeIT without pre-trained	0.6614	0.6582	0.6479	0.6289	0.6617	0.6696	0.6782	0.6573
DeIT without SVM classifier*	0.6561	0.6428	0.6675	0.6708	0.6825	0.6843	0.6638	0.6592
DeiT*^39^	0.6982	0.6820	0.6815	0.7008	0.6963	0.6921	0.7108	0.7034

The test results of our proposed method are shown in bold.

Secondly, for classifier selection in multi-classification problems, most network models default to Softmax, which minimizes cross-entropy from a global perspective. However, the differences between micro-expressions of different sentiment categories are very subtle, and once there are negative samples with large differences, it is likely to lead to misclassification. To solve this problem, the SVM classifier was tried in the experiments. SVM tries to find the maximum margin between different categories of data with better differentiability, it contains a regularization term, penalizes misclassified data more strongly, and has better generalization ability. Similar to the ablation experiments with pre-trained features, we also conducted experiments using different network models, and the final results showed that different models improved the accuracy after using the SVM classifier. Finally, to verify whether the convolutional block attention module and the modified lightweight ViT module can improve the recognition ability of the network, we compare their performance metrics both with the models pre-trained and with the SVM classifier. The table shows that both modules can improve the recognition performance, but the composite model with the addition of the ViT module in the ShuffleNetV2 model achieves better accuracy, on the one hand because the MLP has fewer layers in the CBAM and is applied only once in the network structure, and fewer regions are noticed compared to the improved ViT module. On the other hand, the improved ViT module does not lose any pixel information when encoding the image, so the features obtained are also more global in nature.

In order to demonstrate that our proposed method is still competitive among different methods, we summarize many models including the manual feature extraction method. Since the code of some of the models is not open source and their methods are not tested on the MMEW dataset, the accuracy of their methods on the MMEW dataset is not explicitly written in Table 3. Our proposed method in Table 3 shows a significant improvement in accuracy compared with the hand-extracted feature approach. Deep network models can find subtle and difficult to understand high-dimensional features that are difficult to obtain by hand-extracted features. However, for some complex deep learning networks, the accuracy is still not very high.

**Table 3:** UAR and UF1 performance of different approach under LOSO protocol on different datasets.

Approach	MMEW		CASME II		SMIC		SAMM	
	UAR	UF1	UAR	UF1	UAR	UF1	UAR	UF1
LBP-TOP^10^	0.5628	0.5794	0.7429	0.7026	0.5280	0.2000	0.4102	0.3954
LBP-SIP^13^	0.5208	0.5174	0.5281	0.5369	0.5142	0.4452	0.4169	0.4412
Bi-WOOF^17^	–	–	0.5382	0.7805	–	0.5727	–	0.5211
HOOF^18^	0.5814	0.5982	0.5782	0.5874	0.5696	0.5574	0.5877	0.5639
CapsuleNet^43^	0.7296	0.7115	0.7018	0.7068	0.5877	0.5820	0.5927	0.6520
CNN-LSTM^22^	–	–	0.4125	0.4113	0.4276	0.4150	0.3086	0.3020
NMER^44^	0.3414	0.4253	0.6929	0.7624	0.5555	0.5607	04,894	0.6389
RCN-Best^32^	**–**	**–**	0.6600	0.6584	**0.8131**	**0.8653**	0.6771	**0.7647**
TSCNN^21^	–	–	0.6009	0.6124	0.5924	0.5839	0.6103	0.6083
MobileNetV2^42^	–	**–**	0.6328	0.6125	0.6368	0.6589	0.6236	0.6614
DeiT^39^	0.6921	0.6882	0.6814	**0.6994**	0.6881	0.6970	**0.7052**	0.7028
Proposed method	**0.6981**	**0.7318**	**0.6997**	**0.7251**	**0.7356**	**0.7141**	**0.6781**	**0.7428**

The optimal results of two experiments on different datasets are shown in bold.

On one hand, it is because the number of samples is not enough, and on the other hand, the lightweight neural network framework sacrifices some accuracy to improve efficiency. In traditional deep learning, adequate model prediction results require that the database sample size is large enough and that the training and testing datasets conform to the same distribution. It is clear that the current database does not satisfy these conditions. The table also contains two pure ViT models without convolutional structure, whose accuracy is comparable to that of complex deep networks, but their number of parameters is too large and their training overhead is larger than that of convolutional neural networks. And through Table 3, it can also be found that in the more recently released MMEW dataset, the recognition accuracy has a small improvement compared with other datasets due to its larger number of samples, which laterally reflects that the number of samples plays an important role in the accuracy of micro-expression recognition.

**Figure 7:**
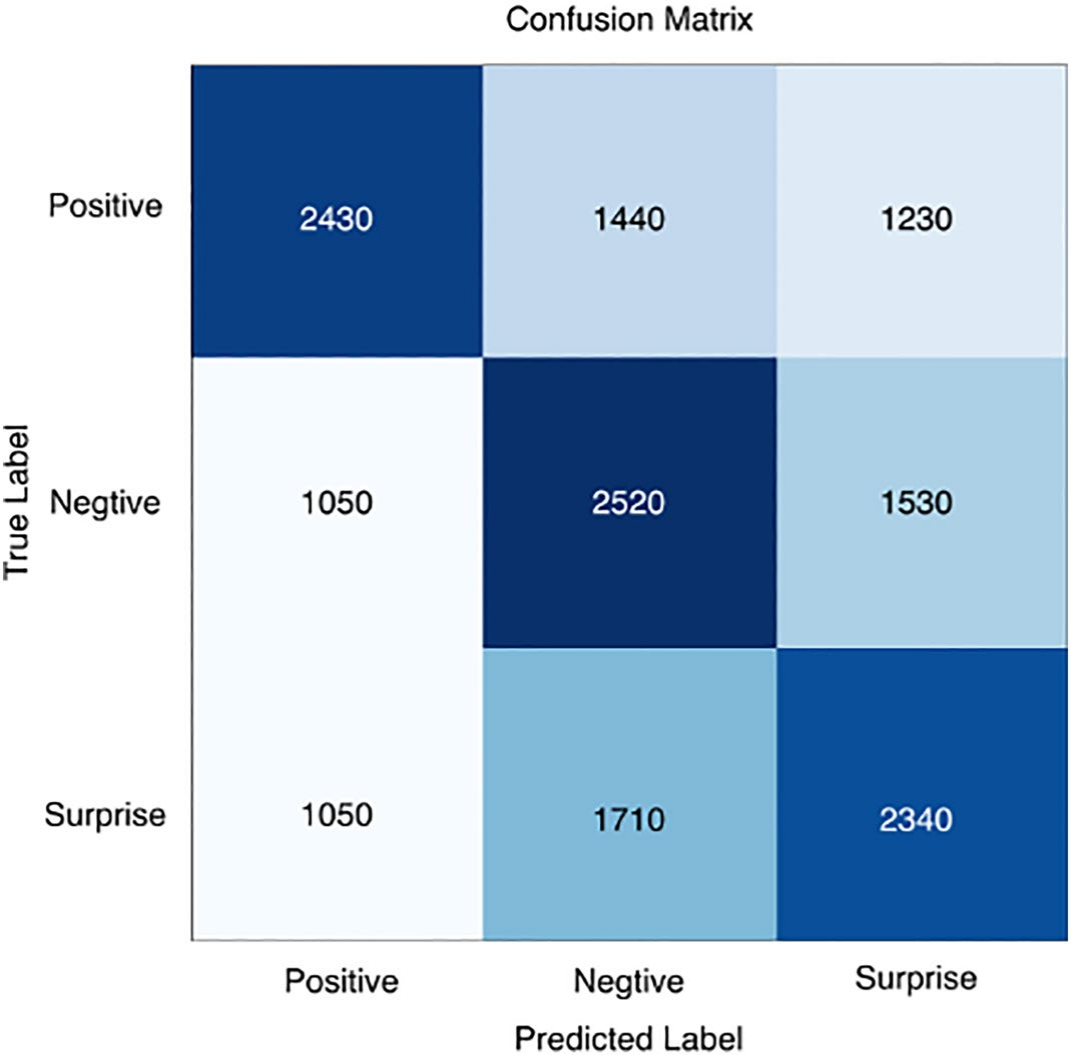
ShuffleNetV2 with CBAM is not pre-trained for optical flow features.

**Figure 8:**
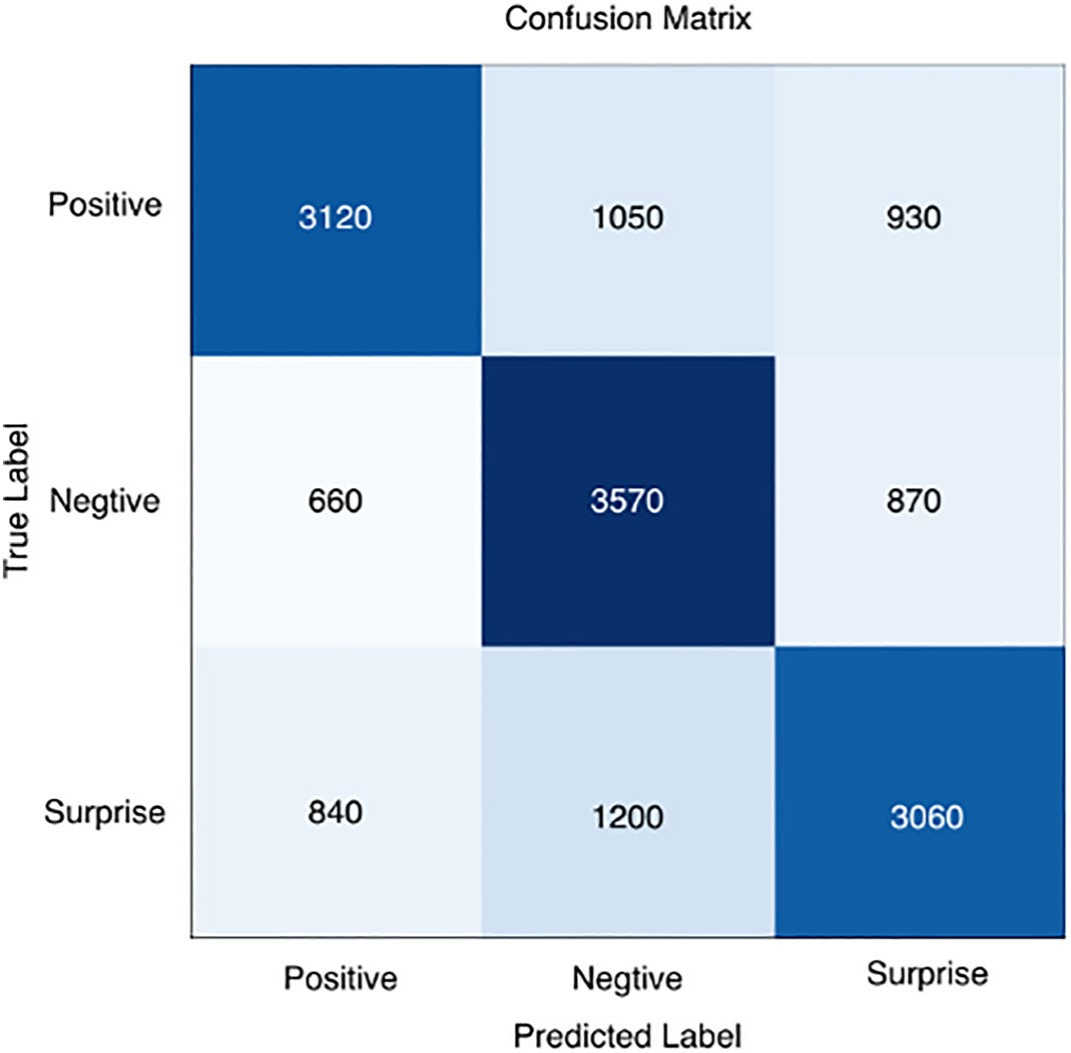
Confusion matrix using ShuffleNetV2 model only.

**Figure 9:**
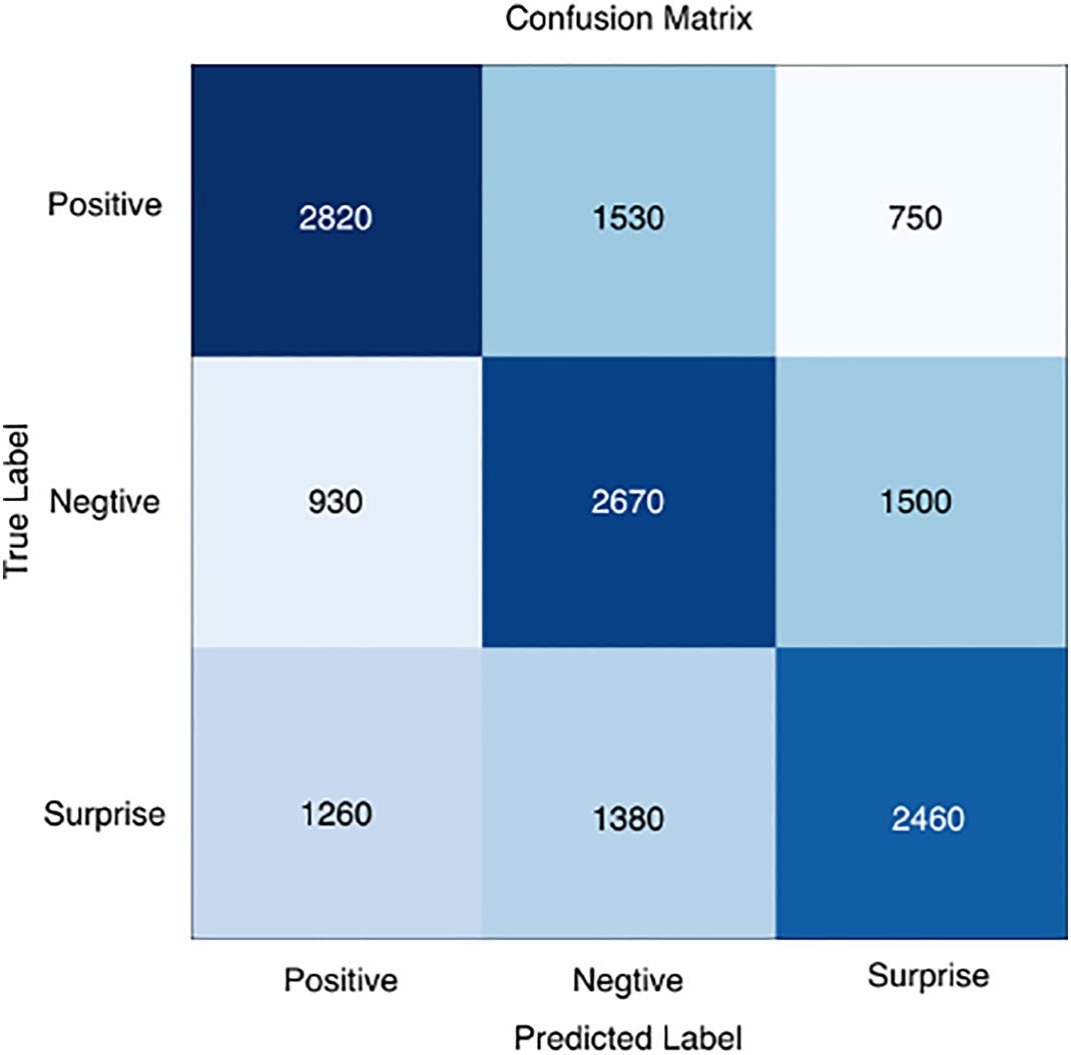
Confusion matrix in the ShuffleNetV2 with CBAM + Softmax case.

**Figure 10:**
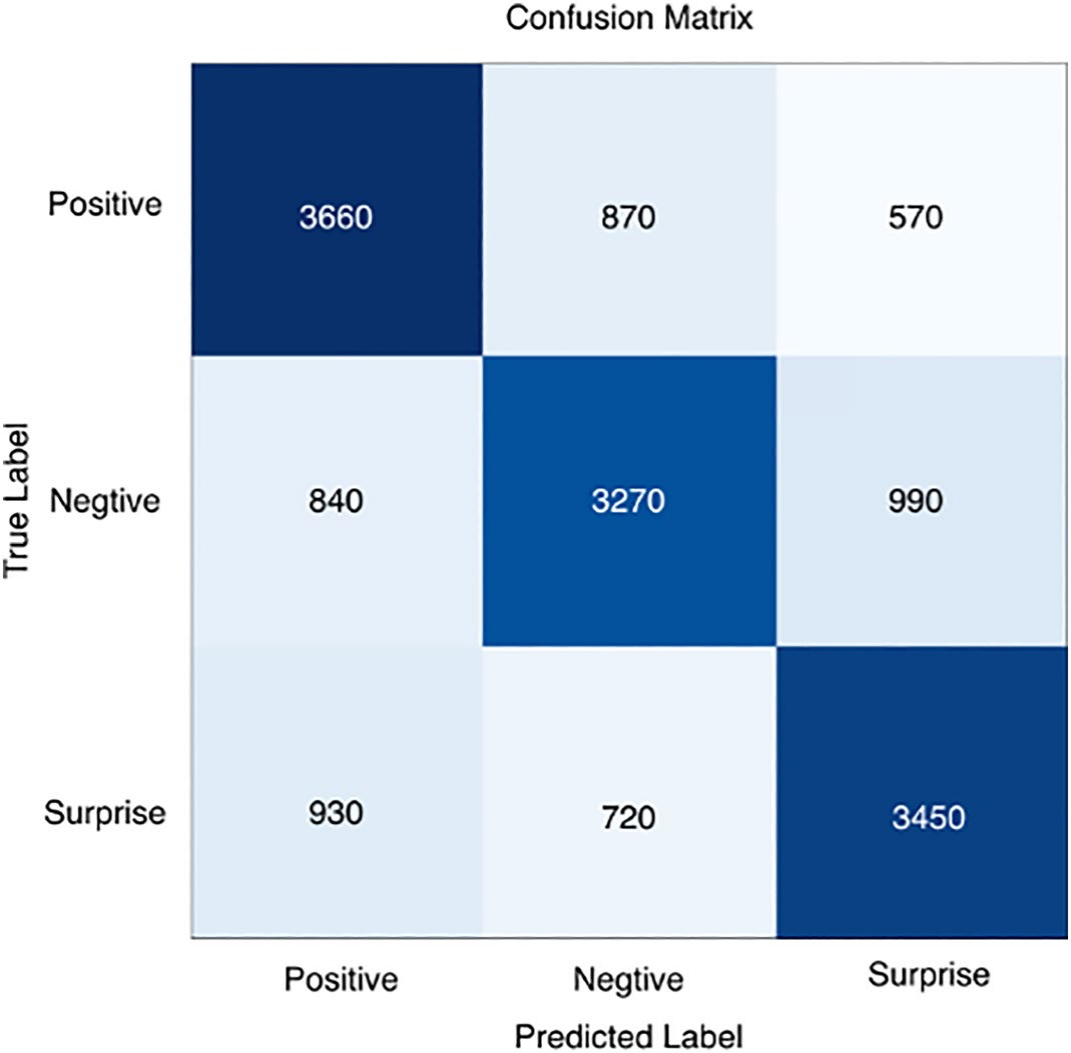
ShuffleNetV2 with CBAM is pre-trained and the classifier uses SVM.

**Figure 11:**
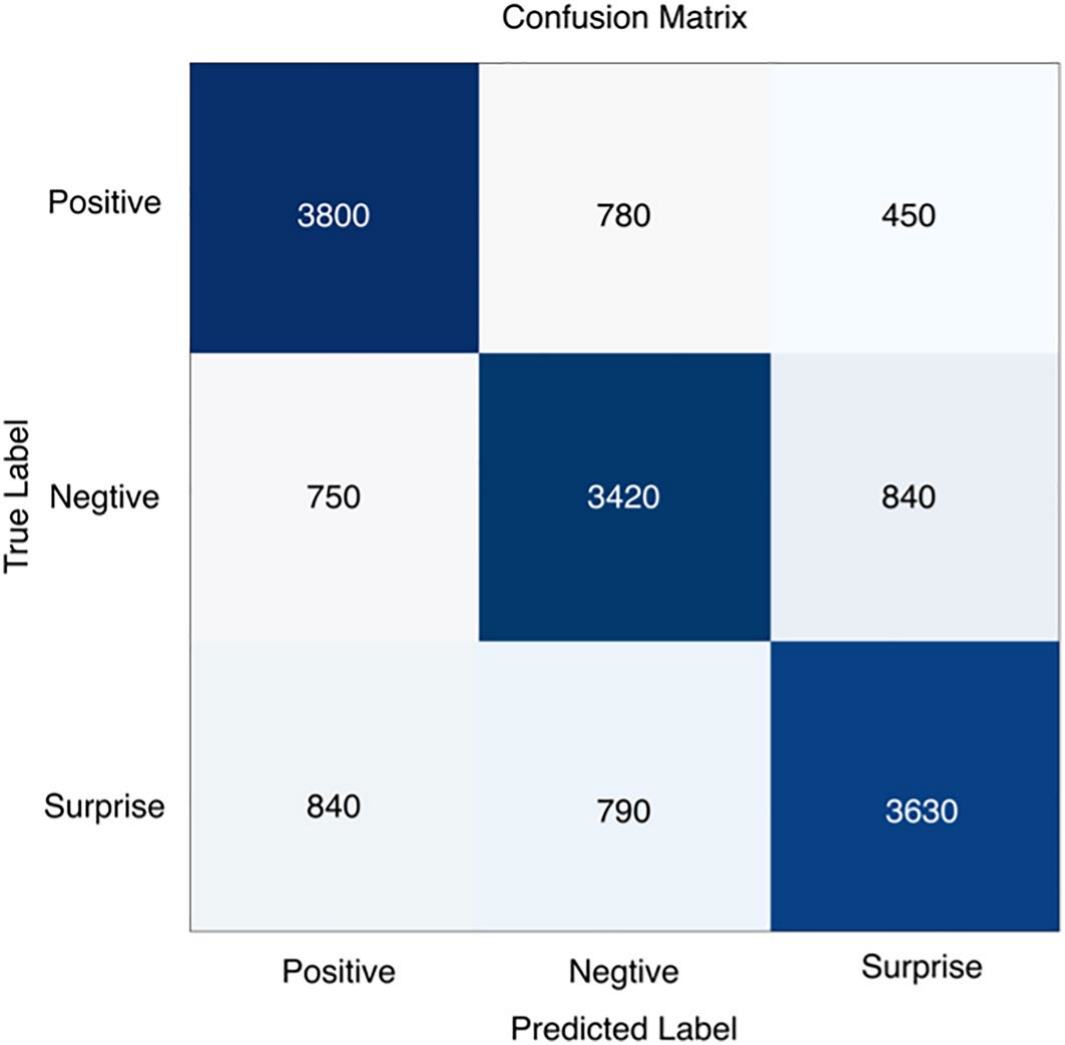
ShuffleNetV2 with improved ViT is pre-trained and the classifier uses SVM.

To analyze in more detail the recognition performance of the three micro-expression categories and the accuracy of the different emotion categories, we calculated confusion matrices, as shown in Figs. 7, 8, 9, 10 and 11, where each confusion matrix is a combination of the three datasets. As can be seen in Fig. 8, the accuracy of positive emotions is the highest compared to negative and surprise emotions. This is because there are only happy samples in the positive category of emotion samples, and the model can grasp their features more easily during the training process. In contrast, the recognition rate of both negative emotions and surprise emotions is low, especially the negative class emotions contain many emotion categories, so it is difficult to obtain accurate features. And the CBAM module with more MLPs compared with the improved ViT module can get the global features better and classify them correctly.

In terms of recognition speed, the experiments compared ResNet50, a conventional CNN, DeiT with no convolutional structure and MobileNetV2, a lightweight CNN, and the average time to recognize an expression under the same experimental environment and equipment is shown in Table 4. Firstly, compared with the complex CNN model, the recognition time of the proposed method in this paper is significantly reduced, which is due to the smaller depth of the network and the smaller number of parameters. In contrast, compared to DeiT, the number of parameters is too large and the training efficiency is much lower than that of conventional CNN, even though its accuracy achieves excellent results over CNN in many fields. Secondly for lightweight CNNs, even the CBAM module, which has a computational overhead close to 0, is slightly behind the lightweight CNN in terms of time, but the improvement in accuracy is necessary for the similar time. Finally, compared with the CBAM module and the improved ViT module, the recognition time of the latter is slightly improved, but there is also a significant improvement in the performance metrics, and the recognition time of both modules is close to the minimum duration of micro-expressions (30 ms), which can capture facial micro-expressions in a timely and effective manner.

**Table 4:** Comparing the time to identify a single sentiment sample by different network models.

Approaches	Time (ms)
ResNet50	105.9
DeiT^39^	168
MobileNetV2^42^	24
ShuffleNetV2 + CBAM	**28.5**
ShuffleNetV2 + ViT	**37.9**

The test results of our proposed method are shown in bold.

The pre-processing part of the data is given great importance in the experiments, and it is demonstrated that the TV-L1 optical flow feature can effectively improve the training accuracy, and the most suitable parameters are determined by trying different expression sequence lengths and facial mask regions. Also, to avoid the problem of unbalanced number of sample categories, the same number of emotion category samples are randomly selected in each training iteration to prevent the neural network model from being more biased towards a certain emotion category. The generalization ability of the model is also improved during the training of the merged dataset, with better stability in the face of new samples. In fact, the facial action area of micro-expressions is very limited in size and contains some irrelevant muscle movements (e.g., blinking) when the face produces an action. Therefore, directly extracting the feature vector of the face using the full-face sample will contain more redundant information, which will reduce the expressiveness of the feature vector and thus affect the recognition accuracy.

When ShuffleNetV2 was proposed, it already surpassed the then state-of-the-art network model in terms of accuracy and efficiency, and it can be seen experimentally that ShuffleNetV2, with the addition of different modules, effectively improves the accuracy of the model on image classification problems. Therefore, the improved ShuffleNetV2 can effectively improve the accuracy on other image classification and recognition problems.

## Conclusion and future work

In this paper, two differently designed self-attentive modules, CBAM and ViT, are proposed to improve the ShuffleNetV2 model, and the model is pre-trained using TV-L1 light-slip features. The final performance in the four datasets is achieved comparable to the best current research and only lags behind the lightweight CNN network in terms of recognition time. The experimental results demonstrate that the proposed two modules can effectively improve the recognition accuracy of ShufleNetV2, which is one of the effective methods for micro-expression recognition. In future work, we will try to extract features containing more information as input to reduce the redundant features while improving the recognition accuracy. In addition, random noise processing is performed on the images to improve the stability of the model and facilitate the recognition of facial micro-expressions in non-ideal environments. Subsequent studies aim to broaden the usage scenarios of micro-expression recognition and reduce the cost of using the network model in order to make micro-expression recognition more useful in different fields.

## Data Availability

The CASME II dataset used to support the results of this study was provided by the Fu Xiaolan research group of the Chinese Academy of Sciences with permission and is therefore not freely available. Requests to access these data should download a signed license file at http://fu.psych.ac.cn/CASME/casme2-en.php and send it to fuxl@psych.ac.cn. The SAMM dataset is provided with permission from the Moi Hoon Yap Research Group at Manchester Metropolitan University and is therefore not freely available. Requests to access these data should be downloaded at http://www2.docm.mmu.ac.uk/STAFF/M.Yap/dataset.php and sent to M.Yap@mmu.ac.uk with a signed permission file. The SMIC dataset is provided by Li Xiaobai's research group at the University of Oulu, Finland with permission, so it is not freely available. To request access to these data, please send an email to Xiaobai.Li@oulu.fi to obtain it. The MMEW dataset used to support the results of this study was provided by the research group of Ben appeared Ye at Shandong University with permission and therefore cannot be provided free of charge. Requests for access to these data should be made by contacting Ben appeared Ye (benxianyeye@163.com) and sending a signed permission file to benxianyeye@163.com.
